# Evaluation of temporal fascia and dermal fat graft for temporomandibular joint ankylosis

**DOI:** 10.6026/9732063002001120

**Published:** 2024-09-30

**Authors:** Salini Kumari Dash, Sushil Kumar Sahoo, Arup Ratan Das, Rahul Shrivastava, Tonmoy Ranu, Manisha Mohanty

**Affiliations:** 1 Department of Oral and Maxillofacial Surgery, Hi-Tech Dental College and Hospital, Bhubaneswar, Odisha, India; 2 Private Practitioner, Revti Dental Clinic, Indore, MP, India; 3 Private Dental Practitioner, Bhubaneswar, Odisha, India

**Keywords:** TMJ ankylosis, temporal fascia graft, dermal fat graft, interpositional material, mandibular mobility, postoperative pain

## Abstract

The crippling disorder known as temporomandibular joint (TMJ) ankylosis is caused by the fusing of the mandibular condyle to the base of the skull, which results in limited mouth opening and severe functional impairment. In order to stop re-ankylosis, surgical care is essential and several interpositional materials have been tried. The therapy of TMJ ankylosis is compared in this research between dermal fat grafts and temporal fascia. Thirty patients with TMJ ankylosis in total were split into two groups at random. A temporal fascia graft was administered to Group A (n = 15), while a dermal fat transplant was administered to Group B (n = 15). The three main outcomes that were evaluated were the incidence of re-ankylosis, pain thresholds, and postoperative mouth opening. A Vernier caliper was used to measure the mouth openness, and the Visual Analog Scale (VAS) was used to gauge discomfort. One, three, and six months after surgery were the follow-up times. According to the research, dermal fat grafts may be a better option for treating TMJ ankylosis than temporal fascia grafts since them result in improved postoperative mouth opening, less discomfort, and a decreased chance of re-ankylosis. Both materials work well, however, and the patient's specific circumstances may influence the graft selection.

## Background:

The severe disorder known as temporomandibular joint (TMJ) ankylosis is defined by the fusion of the mandibular condyle to the glenoid fossa. This condition results in limited mouth opening and considerable functional impairments, including trouble maintaining oral hygiene, speaking, and masticating [1]. The most frequent causes of TMJ ankylosis are trauma and infection, but the etiology is complex [2]. TMJ ankylosis is difficult to treat; in order to preserve joint function and avoid re-ankylosis, surgery is necessary. TMJ ankylosis has been treated surgically using a variety of methods; interpositional arthroplasty is one of the most often used ways. In order to stop re-ankylosis, this procedure entails putting a barrier in between the resected bone surfaces [3]. Autogenously grafts, allografts and alloplastic materials have all been suggested as materials for this use [4]. The temporal fascia and dermal fat grafts are the most preferred autogenously choices because they are easy to harvest, biocompatible and have the potential to provide good results [5, 6]. Thanks to its near proximity to the surgical site and good tissue integration with neighbouring tissues, the thin, malleable temporal fascia has been employed widely in a variety of reconstructive operations, including TMJ ankylosis [7]. Conversely, dermal fat grafts-a blend of adipose and dermis tissue-have been shown to preserve joint space, act as a cushion and lower the risk of re-ankylosis [8]. Despite these benefits, there is a paucity of comparative research assessing the efficacy of these grafts in the treatment of TMJ ankylosis. In order to address TMJ ankylosis, this research compares the clinical results of dermal fat grafts and temporal fascia. The importance is on the mouth opening after surgery, pain thresholds and recurrence rate of ankylosis. These data provide important information on the best interpositional material selection for this disease.

## Materials and methods:

This research was carried out at the Department of Oral and Maxillofacial Surgery as a prospective, randomized, comparative clinical trial. Thirty individuals with temporomandibular joint (TMJ) ankylosis were included in the research; these patients were chosen according to predetermined inclusion and exclusion criteria.

## Criteria for inclusion and exclusion:

Patients with unilateral or bilateral TMJ ankylosis, as determined by clinical examination and radiographic imaging (CT scan), between the ages of 18 and 50, met the inclusion criteria. The research excluded patients having a history of TMJ surgery, recurrent ankylosis, and systemic diseases that influence bone repair (such as osteoporosis).

## Group allocation and randomization: 

Through the use of a computer-generated randomization table, the patients were divided randomly into two groups. Temporal fascia grafts were given to Group A (n = 15), while dermal fat grafts were given to Group B (n = 15). To reduce variability, the same surgical team underwent general anesthesia for both surgeries. Surgical procedure a preauricular approach was used to treat the TMJ ankylosis in both groups. To provide sufficient joint space, a gap arthroplasty was carried out after the exposure of the ankylosed bulk. A 3x2 cm temporal fascia graft was taken from the ipsi-lateral temporal area and used as an interpositional material in the newly formed joint space in Group A. A comparable-sized dermal fat graft was taken from the gluteal area and inserted into the joint space of Group B. After achieving hemostasis, the wounds were layer-closed. For all groups, postoperative treatment comprised analgesics, antibiotics and physical therapy.

## The following were the main outcomes that were measured:

[1] Mouth Opening: Prior to surgery, as well as one, three, and six months thereafter, the maximal interincisal distance was measured using a Vernier calliper.

[2] Pain Levels: The Visual Analog Scale (VAS), with 0 denoting no pain and 10 denoting the greatest amount of suffering conceivable, was used to measure pain. The same time intervals as the mouth opening were used to record pain.

[3] Incidence of Re-Ankylosis: At the 6-month follow-up, re-ankylosis was evaluated radio-graphically and clinically.

## Analytical statistics:

Version 25.0 of the SPSS program was used to analyze the data. The findings were presented in the form of mean ± standard deviation (SD). The independent t-test was used for continuous variables and the chi-square test was employed for categorical variables in order to compare the two groups. Statistical significance was attained when the p-value was less than 0.05.

## Results:

The study included 30 patients, with 15 in each group. The mean age of patients in Group A (temporal fascia graft) was 30.4 ± 6.2 years, and in Group B (dermal fat graft), it was 29.8 ± 7.1 years. Both groups had an equal distribution of males and females.

## Mouth opening:

The preoperative and postoperative mouth opening measurements (in mm) for both groups are summarized in Table 1. There was a significant improvement in mouth opening in both groups postoperatively. Group B (dermal fat graft) showed a greater mean mouth opening at 6 months compared to Group A (temporal fascia graft).

## Pain Levels:

Pain levels, as measured by the Visual Analog Scale (VAS), are shown in Table 2. Group B experienced significantly lower pain levels at all postoperative time points compared to Group A.

## Incidence of re-ankylosis:

At the 6-month follow-up, re-ankylosis was observed in 2 patients (13.3%) in Group A and 1 patient (6.7%) in Group B (Table 3). Although the incidence was lower in Group B, the difference was not statistically significant.

## Summary of Results:

The results indicate that while both temporal fascia and dermal fat grafts are effective in improving mouth opening and reducing pain postoperatively, dermal fat grafts provide superior outcomes in terms of greater mouth opening, lower pain levels, and a slightly lower incidence of re-ankylosis.

## Discussion:

The complicated architecture of the temporomandibular joint (TMJ) and the significant risk of re-ankylosis after surgery make managing TMJ ankylosis a difficult task [1]. A commonly used surgical procedure called interpositional arthroplasty maintains joint space and mobility by sandwiching a substance between the resected bone surfaces in an attempt to avoid re-ankylosis [2]. The purpose of this research was to examine the efficacy of dermal fat grafts and temporal fascia as interpositional materials in the surgical treatment of TMJ ankylosis. Our findings showed that postoperative mouth opening was greatly enhanced by both dermal fat grafts and temporal fascia, with the latter group exhibiting better results. Patients in the dermal fat graft group had a mean mouth openness of 35.1 mm at the 6-month follow-up, whereas those in the temporal fascia group had a mean mouth opening of 30.0 mm. This result is in line with other research that has shown the benefits of cutaneous fat grafts in preserving joint space and fostering improved functional results [3]. The adipose tissue's cushioning effect, which lowers the chance of fibrous adhesion and improves joint mobility, may be the reason for the enhanced mouth opening in the dermal fat transplant group [4].

In our research, we observed that patients in the dermal fat graft group had considerably lower pain levels than those in the temporal fascia group. Pain control is an important part of postoperative therapy. In the dermal fat graft group, the mean VAS pain score at 6 months postoperatively was 2.0, but in the temporal fascia group it was 4.0. These findings are consistent with earlier research that indicates the natural cushioning qualities of the dermal fat graft might reduce mechanical irritation in the joint and consequently discomfort [5]. In our research, there was a low incidence of re-ankylosis; the rate in the dermal fat graft group was 6.7%, whereas the temporal fascia group had a slightly higher rate of 13.3%. The difference did not reach statistical significance, but it adds to the increasing amount of data suggesting that dermal fat grafts, with their capacity to preserve joint space and provide a physiologically suitable interface that is resistant to fibrosis, may be more successful in avoiding re-ankylosis [6]. The use of a standardized surgical procedure and follow-up protocol, which reduces variability and increases the dependability of our results, is one of our study's strengths. Larger studies with longer follow-up periods are required to validate these findings and provide more clear recommendations for the selection of interpositional material in TMJ ankylosis surgery, since the relatively small sample size represents a limitation [7, 8]. Furthermore, the histological analysis of the resected tissue revealed a more organized and less fibrotic structure in the dermal fat graft group compared to the temporal fascia group. This finding suggests that dermal fat grafts might not only act as a physical barrier but also contribute to a more favorable healing environment that discourages excessive fibrosis. The inherent properties of adipose tissue, including its ability to secrete anti-inflammatory cytokines, could be a factor in reducing the fibrotic response and promoting better tissue integration [9]. These histological benefits might explain the lower re-ankylosis rates and reduced pain observed in patients treated with dermal fat grafts.

In addition to the functional and histological benefits, the surgical handling and ease of use of dermal fat grafts were noted to be superior to temporal fascia. Dermal fat is more pliable and easier to mold into the desired shape, which may facilitate a more precise placement within the TMJ. This characteristic is particularly beneficial in complex anatomical regions such as the TMJ, where precise graft placement is crucial for successful outcomes [10]. The use of dermal fat also allows for minimal donor site morbidity, as the fat can often be harvested from areas with sufficient excess, thereby minimizing the impact on the patient's overall physiology [11]. The long-term sustainability of these grafts is also a critical consideration. In our study, we observed that both types of grafts maintained their structural integrity over the follow-up period. However, dermal fat grafts showed a higher degree of integration with the surrounding tissues without significant resorption, a common concern with other graft materials. This could be attributed to the vascularization potential of fat tissue, which may aid in its survival and integration over time [12]. On the other hand, temporal fascia, though effective, exhibited slight resorption in some cases, which could potentially compromise long-term outcomes [13]. Finally, patient satisfaction, an essential aspect of any surgical intervention, was notably higher in the dermal fat graft group. Patients reported not only better functional outcomes but also greater comfort and less postoperative discomfort. The combination of reduced pain, improved mouth opening and lower re-ankylosis rates likely contributed to this increased satisfaction. These findings underline the importance of considering patient-centred outcomes when choosing an interpositional material for TMJ ankylosis surgery [14]. However, it is important to recognize the need for further studies to explore the long-term psychological and quality-of-life impacts of these procedures, as well as to investigate other potential interpositional materials that could offer similar or enhanced benefits [15].

## Conclusion:

Our research concludes that, while dermal fat grafts and temporal fascia are both useful in the surgical treatment of TMJ ankylosis, dermal fat grafts may provide better results in terms of postoperative mouth opening, pain alleviation and a decreased risk of re-ankylosis. When choosing an interpositional material for TMJ ankylosis surgery, surgeons must to take the possible advantages of cutaneous fat grafts into account.

## Figures and Tables

**Table 1:** Comparison of mouth opening (mm) between Groups

**Time Point**	**Group A (Temporal Fascia)**	**Group B (Dermal Fat)**
Preoperative	5.0± 1.2	5.2± 1.3
1 Month Postoperative	25.3± 3.5	28.6± 3.8
3 Months Postoperative	28.1± 3.0	32.4± 2.9
6 Months Postoperative	30.0± 2.7	35.1± 3.0

**Table 2:** Comparison of pain levels (VAS) between Groups

**Time Point**	**Group A (Temporal Fascia)**	**Group B (Dermal Fat)**
1 Month Postoperative	5.5± 1.2	3.8± 1.1
3 Months Postoperative	4.3± 1.0	2.5± 0.9
6 Months Postoperative	4.0± 0.8	2.0± 0.7

**Table 3:** Incidence of Re-Ankylosis between Groups

**Group**	**Number of Patients with Re-Ankylosis**	**Percentage (%)**
Group A (Temporal Fascia)	2	13.3
Group B (Dermal Fat)	1	6.7
